# Clinical Significance of MiR-137 Expression in Patients with Gastric Cancer After Radical Gastrectomy

**DOI:** 10.1371/journal.pone.0142377

**Published:** 2015-11-06

**Authors:** Qiaoyan Gu, Jun Zhang, Haifeng Hu, Yu-e Tan, Shengmei Shi, Yuanyuan Nian

**Affiliations:** 1 Department of Gastroenterology, The Second Affiliated Hospital of Xi'an Jiaotong University, Xi’an, China; 2 Department of Gastroenterology, The Affiliated Hospital of Yan’an University, Yan’an, China; Sapporo Medical University, JAPAN

## Abstract

The dysregulation of miR-137 plays vital roles in the oncogenesis and progression of various types of cancer, but its role in prognosis of gastric cancer patients remains unknown. This study was designed to investigate the expression and prognostic significance of miR-137 in gastric cancer patients after radical gastrectomy. Quantitative real-time PCR (qRT-PCR) was performed to evaluate the expression of miR-137 in human gastric cancer cell lines and tissues in patients with gastric adenocarcinoma. Results were assessed for association with clinical factors and overall survival by using Kaplan-Meier analysis. Prognostic values of miR-137 expression and clinical outcomes were evaluated by Cox regression analysis. The results exhibited that the expression level of miR-137 was decreased in human gastric cancer cell lines and tissues, and down-regulated expression of miR-137 was associated with tumor cell differentiation, N stage, and TNM stage. Decreased miR-137 expression in gastric cancer tissues was positively correlated with poor overall survival of gastric cancer patients. Further multivariate Cox regression analysis suggested that miR-137 expression was an independent prognostic indicator for gastric cancer except for TNM stage. Applying the prognostic value of miR-137 expression to TNM stage III group showed a better risk stratification for overall survival. In conclusion, the results reinforced the critical role for the down-regulated miR-137 expression in gastric cancer and suggested that miR-137 expression could be a prognostic indicator for this disease. In addition, these patients with TNM stage III gastric cancer and low miR-137 expression might need more aggressive postoperative treatment and closer follow-up.

## Introduction

China is one of the countries with the highest incidence of gastric cancer and accounts for over 40% of all new cases worldwide [1,2]. Despite aggressive therapy, the prognosis of advanced gastric cancer in China tends to be dismal [3,4]. At present, tumor-node-metastasis (TNM) staging system of the Union for International Cancer Control/American Joint Committee on Cancer (UICC/AJCC) is the most important prognostic factor for gastric cancer [5]. However, because there is heterogeneity at the molecular level of gastric cancer, it is insufficient to only rely on TNM stage to predict the prognosis of gastric cancer [6]. Therefore, the identification of molecular markers that are predictive of gastric cancer aggressiveness and prognosis of patient as supplementary to TNM stage has the potential to improve the ability to treat patients and could provide clinically relevant insights of importance into disease management. In addition, selecting the patients who might benefit greatest from precision treatment is thought of as the best method to lower the rates of mortality of gastric cancer except for surgery.

MicroRNAs (miRNAs) are small endogenous 19–25 nucleotides non-coding RNAs that regulate gene expression post transcriptionally through base pairing with the 3’-untranslated region (3’-UTR) of target messenger RNAs (mRNAs), that resulting in mRNA degradation or inhibition of translation [7]. In many human cancers, miRNAs can function as oncogenes or tumor suppressor genes to suppress translation or induce mRNA degradation depending on the nature of their targets [8]. Previously studies demonstrated that the expression of miR-137 is down-regulated and considered as a prognostic marker in various types of cancer, including lung cancer [9], colorectal cancer [10], ovarian cancer [11], multiple myeloma [12], gastrointestinal stromal tumor [13], glioblastoma [14], and squamous cell carcinoma of the head and neck [15]. It was recently shown that miR-137 expression was significantly decreased in gastric cancer tissues, and the suppression was concurrently with the severity of pathological changes, suggesting the potential tumor suppressor role of miR-137 in gastric cancer [16]. However, an extensive analysis of expression of miR-137 in correlated to prognosis of gastric cancer patients has not been performed and awaits further elucidation.

The principle aim of this research is to evaluate the relationship between the expression of miR-137 and overall survival of gastric cancer patients in an effort to identify the prognostic significance of miR-137 expression. The results of this study exhibit that the expression level of miR-137 was down-regulation in gastric cancer cells and tissues, and miR-137 expression was an independent prognostic indicator for gastric cancer.

## Materials and Methods

### Ethics statement

This study was reviewed and approved by the Institutional Review Board of The Second Affiliated Hospital of Xi’an Jiaotong University and The Affiliated Hospital of Yan’an University (Shanxi province, China). All study participants, or their legal guardian, provided informed written consent prior to study enrollment.

### Cell culture

Immortalized normal human gastric epithelial cell line GES-1 and five human gastric cancer cell lines, including MKN-45, SGC7901, BGC-823, MGC-803, and AGS, were obtained directly from Shanghai Cell Bank of Chinese Academy of Sciences (Shanghai, China). All these cells were routinely grown and maintained in RPMI-1640 (Gibco) culture medium supplemented with 10% fetal bovine serum (Invitrogen) at 37°C in a humidified cell incubator with an atmosphere of 5% CO_2_.

### Clinical specimens

Fresh gastric adenocarcinoma tissues and adjacent normal tissues were collected from two independent sets comprising 154 patients with gastric adenocarcinoma. All the patients enrolled in this study were underwent radical gastrectomy (R0 resection according to the UICC/AJCC and D2 lymphadenectomy). Specimens of training set (n = 67) were obtained between January 2005 and December 2009 from The Second Affiliated Hospital of Xi’an Jiaotong University (Shanxi province, China), and specimens of validation set (n = 87) were obtained between January 2008 and December 2010 from The Affiliated Hospital of Yan’an University (Shanxi province, China). Samples were flash frozen in liquid nitrogen until use. Specimens were reassessed by two pathologists independently, and the stage of gastric cancer is classified according to the TNM stage system of the UICC/AJCC [5]. Patients were excluded if they had previously been exposed to any targeted therapy, radiotherapy, chemotherapy, and/or intervention therapy for gastric cancer.

### qRT-PCR

Total RNA containing miRNA was extracted from cultured cells or tissues using miRNeasy Mini Kit (Qiagen). cDNA was synthesized using miScript Reverse Transcription Kit (Qiagen) following the manufacturer’s instructions. Reverse transcription was undertaken using 50 ng total RNA with a primer specific for miR-137, together with the SYBR Green microRNA reverse transcription kit. miRNAs were quantified using the SYBR Green miRNA qRT-PCR assay according to the manufacturer’s protocol (Applied BioSystems). The qRT-PCR reaction was carried out on a 7500 Fast Real-time System (Applied Biosystems). All quantitative RT-PCRs were performed in triplicate. The data were analyzed using an automated baseline. The threshold cycle (Ct) was defined as the fractional cycle number at which the fluorescence exceeded the given threshold. The data obtained from the qRT-PCR were analyzed using the ΔΔCt method (2^ΔΔCt^). The PCR primers sets used here for miR-137 was designed as follows: miR-137 forward primer: 5’-GCGCGC TTATTGCTTAAGAATAC-3’, and reverse primer: 5’-GTGCAGGGTCCGAGGT-3’. U6 was used as an internal control and amplified with forward primer: 5’-GCTTCGGCAGCACATATACTAAAAT-3’, and reverse primer: 5’-CGCTTCACGAATTTGCGTGTCAT-3’.

### Statistical analysis

Statistical analysis was performed using GraphPad Prism 5 (GraphPad Software, Inc., San Diego, CA, USA) and SPSS 19.0 (SPSS, Chicago, IL, USA). The overall survival was defined as the time between the first day of diagnosis and the date of cancer-related death or the last follow-up visit. Pearson’s χ^2^ or Fisher’s exact test was used to analyze the relationship between miR-137 expression and clinicopathological factors. Cumulative survival rates were calculated by Kaplan-Meier method and the differences between the subgroups were examined by the log-rank test. Numbers at risk were calculated for the beginning of each time period. The prognostic value of miR-137 expression was determined by univariate and multivariate analysis. All P values were two sided, and P<0.05 was considered to be statistically significant.

## Results

### Expression analysis of miR-137 in human gastric cancer cells

**Fig 1:**
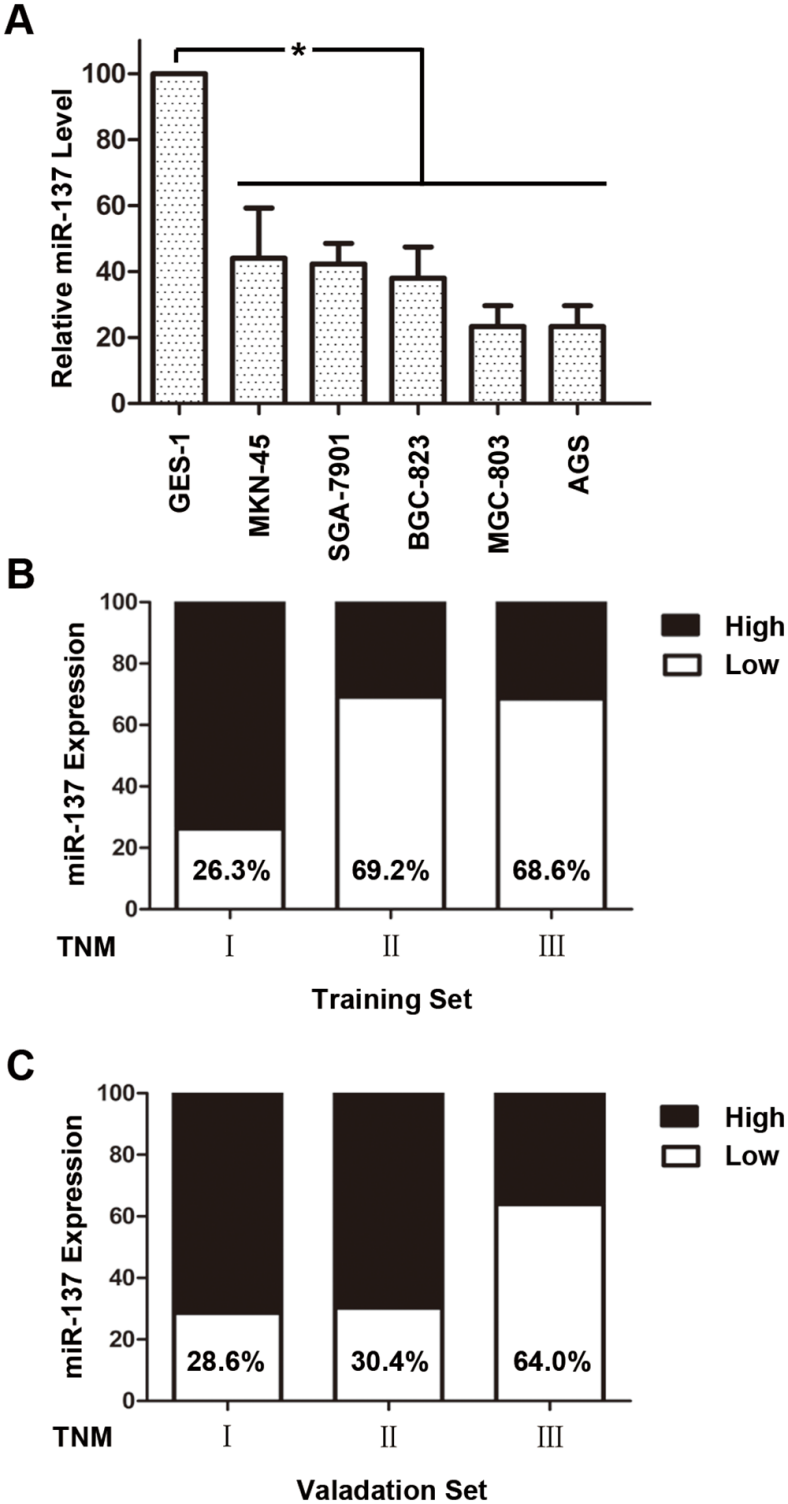
The expression of miR-137 in human gastric cells and tissues. (A) qRT-PCR analysis for miR-137 expression levels in immortalized normal human gastric epithelial cell line GES-1 and five human gastric cancer cell lines (MKN-45, SGC7901, BGC-823, MGC-803, and AGS). (B-C) The percent of patients with low miR-137 expression increased accompanied with disease progression from TNM stage I to III in (B) Training set (n = 67) and (C) Validation set (n = 87). *P<0.05.

In order to ascertain the expression level of miR-137 in human gastric cancer cells, we first evaluated miR-137 expression by qRT-PCR in immortalized normal human gastric epithelial cell line GES-1 and five human gastric cancer cell lines, including MKN-45, SGC7901, BGC-823, MGC-803, and AGS. As shown in Fig 1A, the human gastric cancer cells expressed significantly lower levels of miR-137 than GES-1. Collectively, these observations suggest that miR-137 expression is decreased in gastric cancer cells compared with the immortalized normal human gastric epithelial cell, and decreased miR-137 expression may be related to the oncogenesis of gastric cancer.

### Associations between miR-137 expression and clinicopathologic factors

The expression levels of miR-137 were further measured to analyze their relationship with clinicopathologic factors. Patients were divided into high and low expression group according to the ratio of their normal/cancer tissue median expression levels of miR-137 according the results of qRT-PCR (cut-off ratio = 1.5). And according to the criterion, approximately 56.72% (Training set, 38 of 67) and 49.43% (Validation set, 43 of 87) tumors were scored as low miR-137 expression. As shown in Table 1, the expression of miR-137 was significantly associated with tumor cell differentiation (P = 0.009 and P = 0.002, respectively), N stage (P = 0.019 and P<0.001, respectively), and TNM stage (P = 0.007 and P = 0.007, respectively) in the two independent sets. In addition, in the training set, miR-137 expression was also significantly related with T stage (P = 0.002). Besides, the percent of patients with low miR-137 expression increased accompanied with disease progression from TNM stage I to III in the two independent sets (Fig 1B and 1C).

**Table 1:** Association between miR-137 expression and clinical characteristics.

	Training Set			Validation Set		
	miR-137 Expression			miR-137 Expression		
Factor	High	Low	P	High	Low	P
All patients	29	38		44	43	
Age (years)^†^			0.267			0.458
≤60	22	24		17	20	
>60	7	14		27	23	
Gender			0.260			0.533
Female	12	21		15	12	
Male	17	17		29	31	
Localization			0.817			0.076
Proximal	2	4		13	6	
Middle	14	16		13	22	
Distal	13	18		18	15	
Differentiation			**0.009**			**0.002**
Well	6	1		10	3	
Moderately	14	13		19	9	
Poorly	9	24		15	31	
Lauren classification			0.152			0.132
Intestinal type	23	24		35	28	
Diffuse type	6	14		9	15	
T stage			**0.002**			0.066
T1+T2	17	8		12	5	
T3+T4	12	30		32	38	
N stage			**0.019**			<**0.001**
N0	14	8		24	7	
N1+2+3	15	30		20	36	
TNM stage			**0.007**			**0.007**
I	14	5		10	4	
II	4	9		16	7	
III	11	24		18	32	
Tumor size (cm)^†^			0.057			0.456
<3.5	19	16		19	22	
≥3.5	10	22		25	21	

^†^Split at median.

### Prognostic value of miR-137 expression in patients with gastric cancer

**Fig 2:**
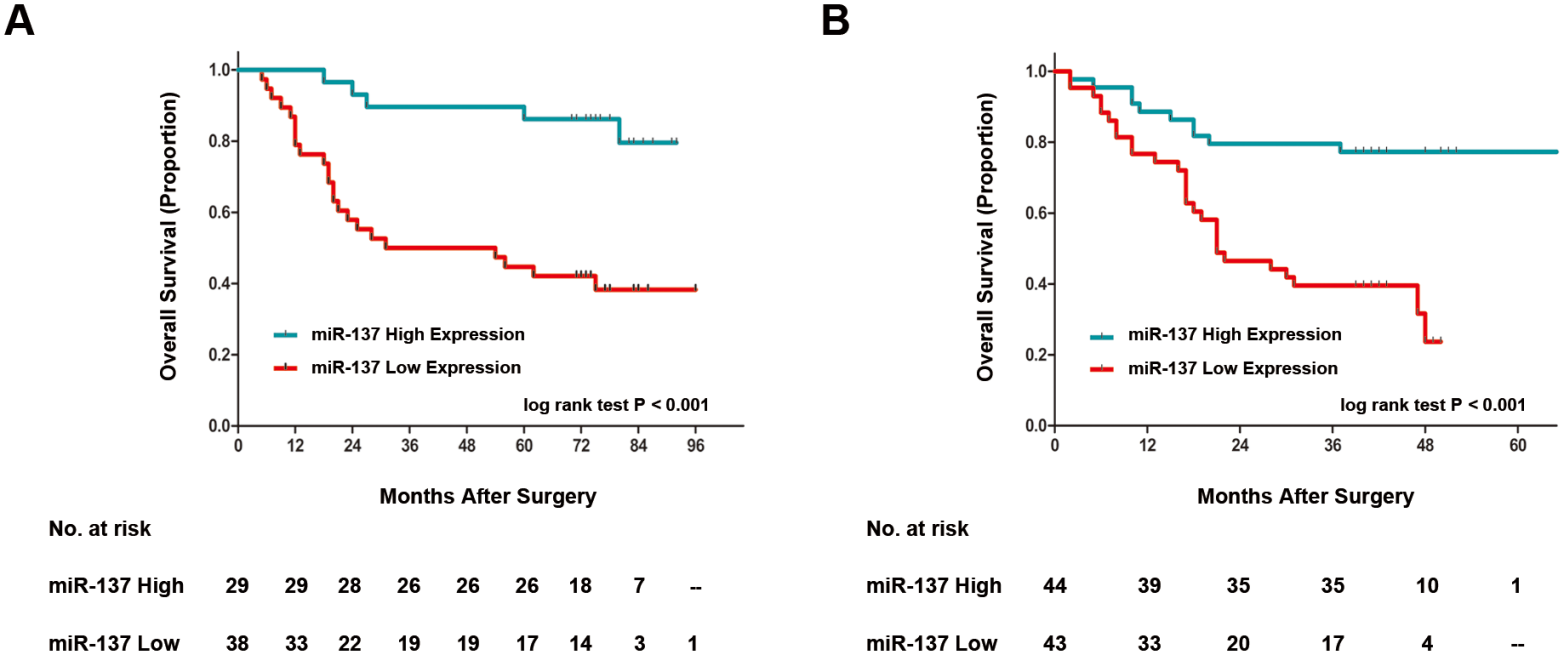
Analyses of overall survival according to the expression of miR-137 in gastric cancer patients. Kaplan-Meier analyses of overall survival according to miR-137 expression in (A) Training set (n = 67) and (B) Validation set (n = 87). P value was calculated by log-rank test.

To further investigate the prognostic value of miR-137 expression in gastric cancer patients, we compared overall survival according to miR-137 expression, and Kaplan-Meier survival analysis was performed. As shown in Fig 2, patients with low miR-137 expression showed significantly shorter overall survival than those high ones in the two independent sets (P<0.001 and P<0.001, respectively), which indicated a vital impact of miR-137 expression on clinical outcome in gastric cancer patients. In addition, univariate analyses for overall survival in this study exhibited that low miR-137 expression is a significant negative prognostic predictor for patients with gastric cancer in the training set (hazard ratio [HR], 6.30; 95% CI, 2.17 to 18.27; P = 0.001) and validation set (HR, 3.74; 95% CI, 1.81 to 7.73; P<0.001). Besides, tumor cell differentiation (P = 0.023 and P = 0.048, respectively), T stage (P = 0.002 and P = 0.012, respectively), N stage (P = 0.004 and P = 0.001, respectively), TNM stage (P<0.001 and P<0.001, respectively) all also significantly affected the survival of gastric cancer (Table 2). Furthermore, Cox multivariate regression analyses were performed to derive independent risk estimates related to overall survival. As shown in the following Table 3, TNM stage and miR-137 expression were both recognized as independent and significant prognostic parameters in the two independent sets (Training set: TNM stage: HR, 12.83; 95% CI, 3.48 to 47.30; P<0.001; miR-137 expression: HR, 6.80; 95% CI, 2.06 to 22.48; P = 0.002; Validation set: TNM stage: HR, 5.21; 95% CI, 2.04 to 13.31; P = 0.001; miR-137 expression: HR, 2.41; 95% CI, 1.13 to 5.11; P = 0.023, respectively).

**Table 2:** Univariate Cox Regression Analyses for Overall Survival.

	Overall Survival			
	Training Set		Validation Set	
Factor	Hazard Ratio (95%CI)	P	Hazard Ratio (95%CI)	P
Age (years)^†^		0.237		0.241
≤60	1.00 (reference)		1.00 (reference)	
>60	1.58 (0.74 to 3.42)		1.50 (0.76 to 2.93)	
Gender		0.199		0.421
Female	1.00 (reference)		1.00 (reference)	
Male	1.65 (0.77 to 3.57)		1.76 (0.89 to 3.48)	
Localization		0.555		0.420
Proximal+ Middle	1.00 (reference)		1.00 (reference)	
Distal	1.26 (0.59 to 2.67)		0.69 (0.48 to 1.87)	
Differentiation		**0.023**		**0.048**
Well+ Moderately	1.00 (reference)		1.00 (reference)	
Poorly	1.59 (1.07 to 2.38)		1.40 (1.00 to 1.95)	
Lauren classification		0.214		0.576
Intestinal type	1.00 (reference)		1.00 (reference)	
Diffuse type	1.62 (0.76 to 3.47)		1.22 (0.61 to 2.47)	
T stage		**0.002**		**0.012**
T1+T2	1.00 (reference)		1.00 (reference)	
T3+T4	23.61 (3.20 to 174.47)		7.78 (1.75 to 23.59)	
N stage		**0.004**		**0.001**
N0	1.00 (reference)		1.00 (reference)	
N1+N2+N3	18.24 (2.47 to 134.64)		4.22 (1.74 to 10.24)	
TNM stage		<**0.001**		<**0.001**
I+II	1.00 (reference)		1.00 (reference)	
III	11.46 (3.43 to 38.28)		6.51 (2.62 to 16.18)	
Tumor size (cm)^†^		0.861		0.089
<3.5	1.00 (reference)		1.00 (reference)	
≥3.5	1.07 (0.50 to 2.28)		1.79 (0.92 to 3.50)	
miR-137 expression		**0.001**		<**0.001**
High	1.00 (reference)		1.00 (reference)	
Low	6.30 (2.17 to 18.27)		3.74 (1.81 to 7.73)	

Abbreviation: 95% CI, 95% confidence interval.

^†^Split at median.

**Table 3:** Multivariate Cox Regression Analyses for Overall Survival.

	Overall Survival			
	Patients		Multivariate	
Factor	No.	%	Hazard Ratio (95%CI)	P
**Training Set**	67	100		
Differentiation				0.234
Well+ Moderately	34	50.7	1.00 (reference)	
Poorly	33	49.3	1.75 (0.46 to 3.61)	
TNM stage				<**0.001**
I+II	32	47.8	1.00 (reference)	
III	35	52.2	12.83 (3.48 to 47.30)	
miR-137 expression				**0.002**
High	29	43.3	1.00 (reference)	
Low	38	56.7	6.80 (2.06 to 22.48)	
**Validation Set**	87	100		
Differentiation				0.270
Well+ Moderately	41	47.1	1.00 (reference)	
Poorly	46	52.9	1.21 (0.86 to 1.70)	
TNM stage				**0.001**
I+II	37	42.5	1.00 (reference)	
III	50	57.5	5.21 (2.04 to 13.31)	
miR-137 expression				**0.023**
High	44	50.6	1.00 (reference)	
Low	43	49.4	2.41 (1.13 to 5.11)	

Abbreviation: 95% CI, 95% confidence interval.

### Stratified analysis on TNM stage

**Fig 3:**
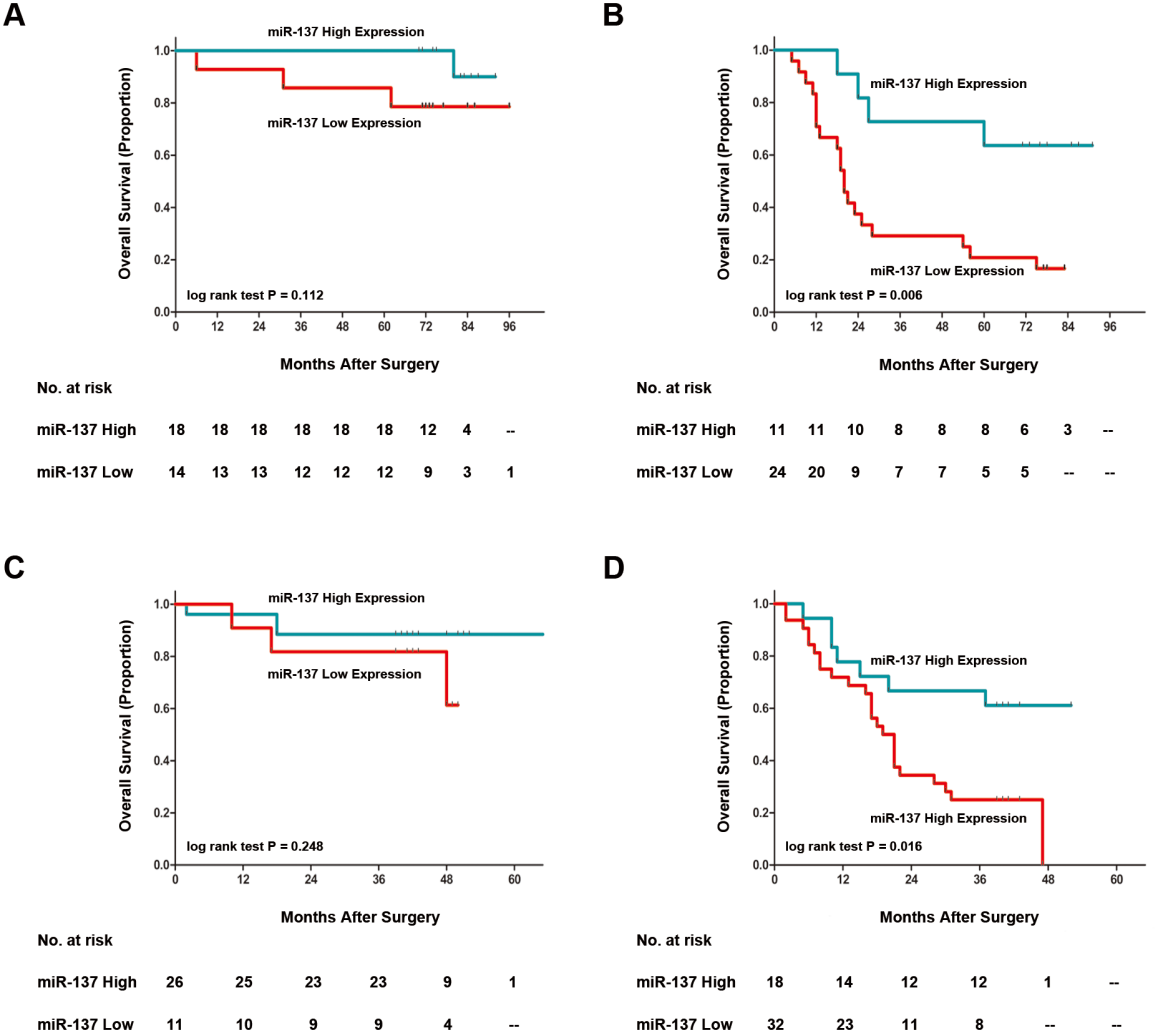
Stratified analysis of overall survival according to the expression of miR-137 in gastric cancer patients with different TNM stage. Kaplan-Meier analyses of overall survival according to miR-137 expression in (A) Training set, TNM stage I+II (n = 32), (B) Training set, TNM stage III (n = 35), (C) Validation set, TNM stage I+II (n = 37), and (D) Validation set, TNM stage III (n = 50). P value was calculated by log-rank test.

To determine whether miR-137 expression could stratify patients with different TNM stage stratum, we did stratified analyses of gastric cancer patients with TNM stage I+II and TNM stage III and evaluated the prognostic value of miR-137 expression in the two independent sets respectively. As listed in Table 4, only the gastric cancer patients with TNM stage III could be stratified by miR-137 expression significantly, the prognosis of TNM stage III gastric cancer patients with high miR-137 expression was significantly better than those with low miR-137 expression in the two independent sets (Training set: P = 0.006, Validation set: P = 0.016, respectively; Fig 3).

**Table 4:** Log-rank test on overall survival for TNM stage split by miR-137 expression.

	Training Set			Validation Set		
Factor	No.	%	P	No.	%	P
All patients	67	100		87	100	
TNM stageI+II	32	47.76		37	42.53	
miR-137 expression			0.112			0.248
High	18	26.87		26	29.89	
Low	14	20.89		11	12.64	
TNM stage III	35	52.24		50	57.47	
miR-137 expression			**0.006**			**0.016**
High	11	16.42		18	20.69	
Low	24	35.82		32	36.78	

## Discussion

China is still one of the countries with the highest incidence of gastric cancer although that in many industrialized nations has decreased during recent decades, and advanced stage disease already present in the vast majority of patients [1,2,4]. Currently, TNM staging system of UICC/AJCC is the most important prognostic factor for gastric cancer [5]. However, because there is heterogeneity at the molecular level of gastric cancer, it is insufficient to only rely on TNM stage to predict the prognosis of gastric cancer [6,17]. Therefore, the identification of molecular markers that are predictive of gastric cancer aggressiveness and prognosis of patient as supplementary to TNM stage has the potential to improve the ability to manage patients and could provide important clinically relevant insights into disease treatment. In addition, selecting the patients who could benefit greatest from precision treatment and targeted therapies is thought of as the best method to reduce the mortality rates of gastric cancer. Research concerning the relationship between miRNAs and gastric cancer has gradually gained attention from researchers in the general surgery field, and this research has become an important research topic in recent years [7]. In the present study, we evaluated the expression level of miR-137 in human gastric cancer cell lines with quantitative real-time PCR, the results exhibited that the human gastric cancer cells, including MKN-45, SGC7901, BGC-823, MGC-803, and AGS, expressed significantly lower levels of miR-137 than GES-1, which is an immortalized normal human gastric epithelial cell line. This result indicated that abnormal miR-137 expression may be related to the oncogenesis of gastric cancer. However, clarifying the underlying mechanism of dysregulation of miR-137 in gastric cancer cells and tissues awaits further investigation.

Same with the other miRNAs, it has been reported that miR-137 was closely correlated with the formation and development of human cancers [8,18]. As important components of gene regulation, in many human cancer cells, miR-137 could regulate different genes and influence their functions in many cellular pathways. Cell proliferation and migration of breast cancer could be suppressed by miR-137 by suppressing estrogen related receptor a (ERRa) [19]. While, in colorectal cancer cells, ectopic miR-137 expression also could arrest cell cycle, repress cell growth, and inhibit cell invasion through targeting cell division control protein 42 homolog (Cdc42) [20]. Recently, Wu et al. indicated that the miR-137 is acted as tumor suppressor on gastric cancer cells by targeting AKT2 and further affecting the Bad and GSK-3β, and potentially involved in tumorigenesis and metastasis of gastric cancer [16]. Zheng and his colleagues demonstrated that ectopic expression of miR-137 was sufficient to inhibit gastric cancer cell proliferation [21]. In addition, Steponaitiene R et al. exhibited that miR-137 methylation is a frequent event in gastrointestinal cancers which occurs early in stepwise manner during gastric carcinogenesis and inversely correlates with global methylation [22]. The above-mentioned studies confirmed that miR-137 plays a role that is similar to tumor suppressor miRNAs. However, all these researches are mainly performed in the gastric cancer cell lines, the relationship between the expression level of miR-137 and the clinical characteristic factors of patients with gastric cancer has not been established. In the present research, we evaluated the association between the expression of miR-137 and prognosis of patients with gastric cancer after radical gastrectomy, and found that miR-137 expression was an independent prognostic factor for overall survival of these patients in addition to TNM stage, and the prognosis of the patients with low miR-137 expression were significantly worse than those with high. To our knowledge, this is the first study to identify miR-137 expression as an independent poor prognostic factor for overall survival of gastric cancer patients following radical gastrectomy. Based on this condition, applying the prognostic value of miR-137 expression to TNM stage III group showed a better risk stratification for overall survival in patients with late-stage gastric cancer. However, the potential changing of clinical practice should be validated in a randomized controlled trial in the future.

To date, since miRNAs represent an emerging hotspot of cancer research, there is an increasing interest with respect to the miRNA responses to environmental exposures and lifestyle, including social status, living conditions, and lifestyle behaviors, such as cigarette smoking and alcohol consumption. Stánitz E et al. showed that miR-21 and miR-143 were up-regulated, and miR-34a was down-regulated in samples of gastric cancer in western countries [23]. In the light of these conditions, whether miR-137 underexpression is also observed in gastric cancer samples of patients from western countries needs to be evaluated in the future. In addition, gastric cancer of the same histopathology from different geographic region's population could show differential miRNAs and protein expression patterns, which might result from the existence of different risk factors of carcinogenesis.

There are several limitations of this study. First, this study is limited by the retrospective nature of the analysis and the selection biases cannot be totally eliminated although we collected the data from a prospectively maintained database. Second, there was heterogeneity in the thresholds used to determine the cutoff of miR-137 expression in this research. The median values were the most consistently used, however a variety of thresholds have been reported. Third, there is not including the data of disease free survival in this study. There are many factors, such as the follow-up examinations and the postoperative treatment, might influence the disease free survival. And the disease free survival data should be collected in the future researches. Finally, the number of patients included in this study is relatively small. Large prospective randomized controlled clinical studies are needed to identify the prognostic value of miR-137 expression in the patients with gastric cancer.

In summary, the results of this study demonstrate that miR-137 is a promising marker to predict the overall survival of patients with gastric cancer after radical gastrectomy. Moreover, these TNM stage III gastric cancer patients with low miR-137 expression might need more aggressive postoperative treatment and closer follow-up.
